# Lifestyle Behaviors Associated with Weight Loss Intent in Adolescent Girls: Findings from the US 2021 National Youth Risk Behavior Survey

**DOI:** 10.3390/nu17101676

**Published:** 2025-05-15

**Authors:** Elinor Fondell, Jaime Vallejos, Andrew J. Piazza, Mariana C. Calle

**Affiliations:** Department of Health Sciences, Worcester State University, Worcester, MA 01602, USAmcalle@worcester.edu (M.C.C.)

**Keywords:** weight loss, weight control, weight dissatisfaction, female adolescents, dietary behaviors, sleep, screen time, physical activity, breakfast, girls’ lifestyle

## Abstract

**Background**: External social influences on body image affect females differently than males, and adolescent girls are more likely to want to change their weight status. Understanding the healthy and unhealthy habits of adolescent girls is vital for developing effective and targeted health promotions and interventions. **Methods**: Using data from the 2021 Youth Risk Behavior Surveillance System (YRBS) survey, this cross-sectional study compares dietary habits, physical activity, vaping, alcohol use, sleep, and screen time in females (9th–12th grades) who intend to lose weight versus those who want to maintain their current weight. **Results**: The sample consisted of 4362 females, of which 56.7% reported an intent to lose weight. The average BMI percentile was 64.1 compared to 75.4 among those trying to lose weight and 50.1 among those not trying to lose weight. Adolescent girls intending to lose weight also reported less frequent breakfast consumption (OR 0.52; 0.40–0.69), less sleep (OR 0.72; 0.59–0.89), more screen time (OR 1.27; 1.02–1.58), engaging in muscle toning exercises (OR 1.30; 1.07–1.57), vaping (OR 1.22; 1.01–1.47), and alcohol use (OR 1.61; 1.32–1.98) compared to those not intending to lose weight. **Conclusions**: Adolescent girls trying to lose weight would likely benefit from interventions to help them improve sleep, reduce screen time, improve dietary and exercise habits, and monitor alcohol and vaping use.

## 1. Introduction

Obesity prevalence reached 22.2% among adolescents aged 12–19, based on the latest National Health Report released in 2021 [1]. Around 80% of obese adolescents carry it into adulthood, leading to health risks such as metabolic tissue dysfunction, low-grade systemic inflammation, cardiovascular diseases, and type 2 diabetes [2,3].

External social influences on body image affect females differently than males [4]. Weight dissatisfaction is particularly prevalent among girls, ranging from 19.2 to 83.4% among 10–19-year-old girls in an international systematic review [5]. Perception of body weight plays a role in the motivation to lose weight in young adults, and physically active adolescents have reported a better body perception [6,7]. However, there is less information regarding physical activity in adolescent girls intending to lose weight. The PA Guidelines for Americans recommend 60 min (1 h) or more of moderate-to-vigorous physical activity daily, including aerobic, muscle-strengthening and bone-strengthening exercise for children and adolescents [8]. Unfortunately the adherence to these recommendations in adolescents is low, approximately 23.9% adherence overall, and significantly lower for girls at 15.7% [9].

The Healthy Eating Index (HEI) score for boys and girls aged 14–18 is 51/100 [10]. The average vegetable intake for girls that age is less than 1 serving per day. Adhering to the Dietary Guidelines for Americans increases the likelihood of maintaining body weight since it encompasses balance, moderation, variety, and calorie control.

The American Academy of Sleep Medicine recommends that teenagers aged 13 to 18 sleep 8 to 10 h per 24 h regularly to promote optimal health [11]. There is a consistent association between screen time and sleep in adolescents [12]. Data from the longitudinal, U.S.-based Adolescent Brain Cognitive Development (ABCD) study showed that females recorded about 1 h more average daily app and smartphone use than males [13]. Coincidentally, the Centers for Disease Control and Prevention (CDC) reported that the percentage of high school students who do not get enough sleep is higher among girls (80%) compared to boys (75%) [14]. Furthermore, inadequate sleep (including both short duration and poor quality) is associated with overweight and obesity in adolescents [15]. Adolescents are also susceptible to direct pulmonary injury, addiction, and other health risks from e-cigarettes [16]. According to the Monitoring the Future survey report, by the National Institute of Health, Institute on Drug Abuse, 27% of 12th grade students had vaped nicotine within the last 12 months in 2022, while 52% of students had used alcohol within the last 12 months in 2022 [17].

While there have been some descriptive studies on adolescents’ habits [9,13,18,19,20], to date, a comprehensive analysis of lifestyle habits among girls intending to lose weight using a representative US sample has been limited [21,22,23].

Understanding the health-related behavior of girls intending to reduce their weight can inform strategies for addressing body weight management in this population. Using a national representative sample, this cross-sectional study compares dietary habits, physical activity, vaping, alcohol use, sleep, and screen time in females (9th–12th grades) who intend to lose weight versus those who want to maintain their current weight. Additionally, this study describes the relationship between actual BMI (using self-reported weight) and weight perception between the two groups of girls (weight loss intent vs. maintaining weight).

## 2. Materials and Methods

### 2.1. Study Design

The 2021 Youth Risk Behavior Survey (YRBS) is part of a biennial cross-sectional survey conducted by the CDC since 1991 to monitor the prevalence of health risk behaviors among 9th through 12th grade students in public and private schools in the United States. YRBS utilizes a three-stage cluster sample design to generate a representative sample. Each student record was assigned a weighting factor based on student sex, race, ethnicity, and grade to adjust for nonresponse and the oversampling of black and Hispanic students in the sample. The weighted count of students equals the total sample size, and the weighted proportions match the national population proportions.

The 2021 YRBS covered 152 schools, received 17,232 usable questionnaires, and had an overall response rate of 57.5% (72.7% school response rate and 79.1% student response rate) [24]. Of the 87 questions covered in the questionnaire, only 58 were required, and schools could customize the survey. Question Q67 regarding weight loss was an optional question and was completed by 9273 students, of which 4362 were girls. The test–retest reliability has been shown to be strong for the questions in YRBS [25]. The institutional review board at the CDC approved the protocol for the YRBS. The YRBS has been described in more detail here [24]. In addition, the present authors obtained IRB approval at their institution to conduct this secondary data analysis.

### 2.2. YRBS Measures

The dependent variable, intent to lose weight, was measured as “Which of the following are you trying to do about your weight” (lose weight, gain weight, stay the same weight, I am not trying to do anything about my weight). For the main analyses, this question was dichotomized into (trying to lose weight, not trying to lose weight).

A healthy diet was measured by looking at vegetable intake, fruit intake, breakfast consumption, soda, and milk intake. Dichotomized versions were used for the variables fruit, soda, milk, and breakfast consumption (yes or no regarding 1 or more fruit per day during the past 7 days, 1 or more soda [not including diet soda] per day during the last 7 days, 1 or more glass of milk per day during the previous 7 days, eating breakfast on all 7 days before the survey).

We used two measures to assess physical activity: muscle strengthening (“During the past 7 days, on how many days did you exercise to strengthen or tone your muscles, such as push-ups, sit-ups, or weight lifting?”) and being physically active for 60 min per day (“During the past 7 days, on how many days were you physically active for a total of at least 60 min per day?”). Screen time was measured as time spent in front of a TV, computer, smartphone, or other electronic device watching shows or videos, playing games, accessing the Internet, or using social media on an average school day, not counting time spent doing schoolwork. This variable was dichotomized into spending 3 or more hours per day on screen time (yes, no). Sleep was dichotomized into getting 8 h or more of sleep on an average school night (yes, no). Alcohol use was dichotomized as drinking at least one drink on at least 1 day during the last 30 days before the survey (yes, no). Similarly, vaping was assessed as using electronic vapor products (including e-cigarettes, vapes, vape pens, e-cigars, e-hookahs, hookah pens, and mods) on at least 1 day during the 30 days before the survey. The BMI percentile was available in the YRBS data set and calculated using the 2000 CDC growth charts [26].

Body size perception was derived from a question regarding how they view their weight (very underweight, slightly underweight, about the right weight, slightly overweight, very overweight) and comparing it to their BMI percentile (overweight/obese ≥ 85th BMI percentile). Body size perception was then categorized into underestimator (those who perceive themselves lighter than their actual weight), accurate estimator (their weight perception is close to their actual weight classification), and overestimator (their body weight perception is above their actual weight).

### 2.3. Data Analysis

All variables were weighted to adjust for nonresponse and oversampling. SPSS complex samples were used to account for the YRBS survey design and weighting. Logistic regression was used to assess the association between dietary variables, physical activity variables, sleep, screen time, alcohol intake, vaping, BMI percentile, and weight loss intent. The fit of the model was assessed by examining ROC curves (AUC = 0.78), which indicated a good fit and good discrimination between groups.

To assess whether associations differed among girls with good versus poor body perception, we conducted analyses stratified by body size perception.

SPSS v29 was used for all statistical analyses (IBM Corp. Released 2023. IBM SPSS Statistics for Windows, Version 29.0.2.0 Armonk, NY, USA: IBM Corp). Statistical significance was determined at the 5% level (2-sided).

## 3. Results

### 3.1. Participant Characteristics

The mean BMI percentile among the girls in our sample was 64.2 (95% CI 62.4–65.9). In total, 55.5% (95% CI 53.6–57.4) wanted to lose weight. This intent was the highest among girls of Hispanic (66.3%) or multiple-Hispanic descent (59.9%) and lowest among girls of Asian descent (50.5%) or ‘Other’ descent (50.0%) (Table 1).

### 3.2. Weight Loss Intent and Lifestyle Behaviors

Results from the weighted logistic regression models are presented in Table 2 and were adjusted for race/ethnicity, grade level, BMI percentile, vegetable consumption, muscle strengthening, sleep, screen time, alcohol intake, and vaping. Girls trying to lose weight were more likely to report engaging in muscle-strengthening exercises (OR 1.30, 95% CI 1.07–1.57), spending 3 h or more per day using screens (OR 1.27; 1.02–1.58), drinking alcohol (OR 1.61; 1.32–1.98), and vaping (OR 1.22; 1.01–1.47). Girls trying to lose weight were less likely to report eating breakfast daily (OR 0.52; 0.40–0.69), eating other vegetables (not including potatoes, salad, or carrots) (OR 0.78; 0.67–0.90), and getting 8 h of sleep (OR 0.72; 0.59–0.89) (Table 2).

### 3.3. Weight Loss Intent and BMI

**Figure 1:**
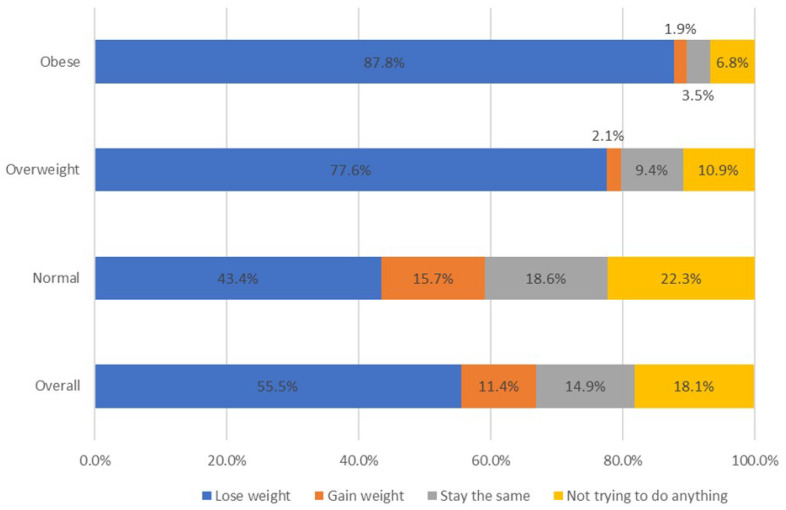
Proportion of adolescent girls reporting weight loss intent by BMI percentile categories (Normal Weight, Overweight, Obese) in the 2021 YRBS Survey: BMI, Body Mass Index. Normal weight BMI% < 85, overweight BMI% 85–<95, obese BMI% ≥ 95.

A large proportion of girls within the normal weight category indicated an intent to lose weight despite being within the normal BMI percentile (43.4%). The intent to lose weight was the highest among obese girls (87.8%). (Figure 1) As previously shown in Table 1, Hispanic and multiple-Hispanic girls were more likely to want to lose weight when considering the entire sample of girls. When considering girls within normal weight only, this body size dissatisfaction was the largest among white girls and girls of Asian descent (47.5% and 45.7%, respectively).

## 4. Discussion

This study examined the associations between sleep, screen time, physical activity, dietary-related variables, alcohol, vaping, and weight loss intent among adolescent girls. The relationship between BMI percentile and weight loss intent is also described. We found that girls intending to lose weight were associated with several unhealthy lifestyle behaviors. Girls intending to lose weight were more likely to report skipping breakfast, more screen time, and less sleep. Furthermore, a weight loss intent was associated with a higher consumption of alcohol and vaping use. The only healthy lifestyle behavior associated with weight loss intent was muscle-strengthening exercises. Due to the cross-sectional nature of the data, we cannot know if girls engaging in these unhealthy lifestyle activities are more likely to develop an intent to lose weight or if girls intending to lose weight are more likely to develop listed unhealthy lifestyle habits. Lastly, we found that weight-loss intent was most common among obese girls. However, many girls within a normal BMI range also reported an intent to lose weight.

### 4.1. Physical Activity

There were no differences in following the PA recommendation of 60 min. per day among the groups. Our results show that girls trying to lose weight were more likely to do strength training 1–3 times per week. These results were similar to data from the 2017 YRBS [22]. Engaging in muscle-strengthening activities can increase body weight, making it harder to fit the slim female ideal often seen in the media. Therefore, it is possible that girls who engage in strength training do so with the intention of losing weight, rather than the training itself leading to weight loss.

Physical activity can help regulate body weight [27] and dramatically lower the risk of many chronic illnesses [28]. Promoting increased physical activity levels could improve health in all adolescents regardless of weight loss intent. Conversely, the correlation between weight loss intent and muscle-strengthening exercise should be studied further to identify potential needs to improve body image among girls in sports. Low energy availability is highly prevalent among female athletes, up to 90% in some sports like ballet [29]. It is crucial to ensure proper food intake in these girls because under-fueling in sports can not only reduce sports performance but also impair immune function, bone health, cardiovascular health, and menstrual function [29].

### 4.2. Diet

In the present study, those trying to lose weight were less likely to eat other vegetables than those who were not trying to lose weight. Other vegetables refer to any vegetable, excluding potatoes, carrots, and salad greens. The YRBS survey only includes questions on the frequency of consumption of fruits and vegetables, and it does not specify the amounts. Although we did not see any significant differences in the consumption frequency of fruits and vegetables, aside from other vegetables, we could not explore a difference in the portion sizes of fruits and vegetables consumed.

Intervention studies targeting increased vegetable intake in this group can improve eating behaviors. However, as previously stated, the frequency of fruit and vegetable intake was low across this population, indicating that both groups of girls would benefit from increasing fruit and vegetable consumption. Adolescents have limited freedom to choose their diet as they follow their family’s eating habits and grocery shopping. In this context, school cafeterias could play a pivotal role in offering healthier options to provide opportunities to increase intake.

Our results indicate that skipping breakfast was correlated with a desire to lose weight, confirming results from a previous study [21]. Skipping breakfast has also been positively associated with obesity [30] and negative body image [31] in girls. The association between breakfast and weight can also be attributed to other factors, such as sleep deprivation. According to data from Project EAT (Eating and Activity in Teens and Young Adults) [18], those who went to bed after 12:30 a.m. were more likely to skip breakfast.

A crossover study using block randomization of breakfast type in children showed that low-glycemic index foods eaten at breakfast had a significant impact on food intake at lunch [32]. Consuming breakfast increases satiety and contributes to higher fiber intake in adolescent girls [33]. Common barriers expressed by adolescents to have breakfast are lack of time and not being hungry [21]. This could be associated with eating late, which is usually associated with being awake late at night. Early school start times are also a likely contributor to poor breakfast habits and delaying school start time has been shown to improve breakfast consumption among highschoolers [34]. Insufficient sleep changes melatonin levels that in turn affects energy homeostasis related to the melanocortin system in the hypothalamus [35]. Additionally, sleeping duration regulates ghrelin, a hormone that stimulates appetite and feeding behavior [36].

Given the health consequences of skipping breakfast, providing adolescents with strategies to incorporate breakfast in their daily routine is paramount. An environment conducive to healthy food choices is key to implementing effective behavioral changes. An example is to provide incentives for participation in the school breakfast program, offering high-quality nutrients and breakfast options that are low in saturated fat and sugar. Education is only one aspect of health promotion. A more comprehensive approach needs to be applied to change health behaviors.

### 4.3. Sleep and Screen Time

Sleep is another lifestyle factor affecting body weight. Adolescents are chronically sleep-deprived [37]. The percentage of high school students who do not get enough sleep is higher among girls (80%) [24]. Regularly sleeping fewer than the recommended hours is associated with attention, behavior, and learning problems [11]. In the present study, girls trying to lose weight were less likely to sleep 8 h or more. Lack of sleep in adolescents affects their cognitive functioning and mental health. A recent review on sleep deprivation reported associations between sleepiness and subjective perception of depression, anxiety, and antisocial behavior [38]. Improvements in sleep need to be addressed in all adolescents, but specific strategies could be tailored to girls trying to lose weight.

Screen time also affects sleep [39]. The percentage of girls spending more than 3 h on screen time was high overall, and it was higher in those girls who intend to lose weight (81%) versus those who do not (75%). These results are consistent with reported data on screen time and body weight dissatisfaction [23], and girls who want to lose weight are more likely to spend more than 3 h per day using screens (not counting school work). Consistently, girls with higher than median screen time over a two-year period rated their body image lower than girls with below median screen time screen [40]. These studies showed the negative role of screen time on body image. The association between screen time and suicidality was 4.67% mediated by overweight/obesity (observed only in female adolescents) and 9.66% mediated by self-perceived overweight (both male and females) [41]. There is likely a bidirectional association between screen time and weight loss intent. Reducing social media use has been shown to improve body satisfaction in adolescents [42]. Therefore, limiting screen time in girls may be highly important to improve mental health and body image. A 7-month school-based intervention using social cognitive theory reduced screen time in overweight and obese adolescent girls [43]. Results from a meta-analysis showed that interventions targeting screen time are effective in reducing total screen time and television time in children and adolescents [44]. Hence, the implementation of these interventions in this group of girls is possible and warranted.

### 4.4. Vaping and Alcohol Use

Preventing primary use in teens and young adults is the most important step that can be taken to reduce the long-term complications of nicotine exposure [16]. In the present analysis, girls who intend to lose weight are more likely to vape. Consistent with our results, Mohapatra et al. reported in their recent systematic review that the high rates of vaping seemed to correlate with increased weight concerns, particularly among females. Girls facing body image pressures may see vaping as a weight loss or weight control strategy [45].

Alcohol intake was higher in females intending to lose weight in this sample. These results are consistent with those from the COMPASS, which is a longitudinal cohort study of secondary school students (Grades 9–12) in Canada [46]. Restrictive eating behavior has also been associated with binge drinking [47], suggesting that girls intending to lose weight may be more prone alcohol consumption. Risky behaviors, such as vaping and drinking alcohol, may be used as coping mechanisms [48]. Therefore, this group may benefit from increased support from school counselors to address mental health and prevent addictive behaviors.

Overall, vaping and alcohol are public health issues that need to be addressed in youth and understanding motivation can help to better target prevention strategies. Existing campaigns, such as the U.S. Food and Drug Administration’s (FDA’s) Real Cost Campaign [49], have been shown to influence youth beliefs related to vaping and can be useful tools to help students avoid or quit vaping [50]. Tackling one of the potential factors triggering these addictive behaviors can be a cost-effective measure. Thus, offering programs targeting girls promoting healthier strategies to manage and accept their body weight is an appropriate preventative measure.

### 4.5. BMI and Weight Loss Intent

Despite having a normal BMI, more than half of these girls are actively trying to change their weight—revealing a significant degree of body image dissatisfaction. Notably, 43% are attempting to lose weight, while 16% are striving to gain weight.

This corresponds well with previous years of YRBS. Approximately 80% of the girls within BMI of 85–95% intend to lose weight in our sample; this is consistent with another representative sample of American adolescents where 86.9% of the ones in the overweight category reported that they intend to lose weight [51].

Culture impacts body image perception. Additionally, the immediate environment, such as family and friends, plays a role in how adolescents perceive themselves. A recent study reported that frequent negative familial weight talk was associated with higher weight bias internalization across gender in non-Hispanic White 10–15-year-old children living in MA [52]. In the present analysis, weight loss intent was the highest among white girls and girls of Asian descent. Similarly, according to data from The Ningbo Youth Risk Behavior Survey, self-perception of overweight and obesity was positively associated with lower-calorie diets and increased levels of PA in Chinese adolescents [53]. This information can be used to specifically target more vulnerable girls and develop culturally appropriate programs for these groups.

### 4.6. Limitations

The general limitations of the 2021 YRBS survey were previously reported [24]. Limitations specific to this study are outlined below. Given the cross-sectional nature of the YRBS, it is impossible to determine the temporal directionality of associations or assess the directionality of correlations. Future studies should use a longitudinal design to establish cause-and-effect relationships. Nevertheless, cross-sectional research can help identify subpopulations that may be more vulnerable and help provide targeted interventions. As mentioned earlier, the YRBS survey includes questions on the frequency of food consumption, not specific amounts. This limited our analyses and we were unable to assess portion sizes. All questions in YRBS were assessed through self-reports, which can lead to overestimation or underestimation of the variables measured. The 2021 survey was conducted during COVID-19, which may have influenced dietary behaviors and access to food. Despite any limitations COVID-19 might have introduced, it will be useful to have these results recorded in the literature so comparisons can be made as future versions of the YRBS are administered.

## 5. Conclusions

School interventions may focus on offering healthier lunch options and providing breakfast programs, emphasizing high-quality nutrients that can enhance eating habits among all youth. Parents and caregivers should limit screen time to promote better sleep patterns, improve mental health, and foster positive body image. Programs designed to prevent substance abuse among youth should specifically target girls who are attempting to lose weight, as they are at a higher risk.

## Figures and Tables

**Table 1:** Demographic characteristics of adolescent girls by weight loss intent status (2021 YRBS).

	Total		Trying to Lose Weight		Not Trying to Lose Weight	
	Unweighted *N*	Weighted % (95% CI) Mean (SE)	Unweighted *N*	Weighted % (95% CI) Mean (SE)	Unweighted *N*	Weighted % (95% CI) Mean (SE)
Age						
<14 years old	819	20.4% (18.8–22.1)	448	20.2% (18.1–22.4)	371	20.7% (19.1–22.4)
15–16 years old	2192	49.8% (48.4–51.2)	1229	50.2% (48.2–52.1)	963	49.4% (46.6–52.1)
>17 years old	1347	29.5% (28.3–31.3)	769	29.7% (27.6–31.8)	578	30.0% (27.4–32.7)
Grade						
9th	1058	24.8% (23.4–26.2)	586	24.9% (23.0–26.9)	472	24.6% (22.9–26.7)
10th	1127	25.2% (23.2–27.3)	619	24.6% (22.1–27.2)	508	26.0% (23.5–28.7)
11th	1068	25.5% (23.9–27.2)	595	25.4% (22.6–28.5)	473	25.6% (23.3–28.1)
12th	1102	24.5% (23.1–25.9)	644	24.9% (23.2–26.7)	458	23.9% (21.6–26.5)
Race/Ethnicity						
Asian	259	5.6% (3.2–9.8)	130	5.0% (3.0–8.1)	129	6.4% (3.3–12.3)
Black	713	12.2% (8.6–16.9)	372	11.4% (7.9–16.3)	341	13.1% (9.4–18.0)
White	2033	48.5% (43.4–53.7)	1142	48.2% (43.2–53.1)	891	49.0% (43.1–54.9)
Hispanic	346	9.8% 7.2–13.2)	227	11.4% (8.3–15.5)	119	7.7% (5.7–10.2)
Multiple Hispanic	637	17.1% (14.9–19.5)	383	18.0% (15.8–20.5)	254	15.9% (13.1–19.1)
Other (American Indian, Alaska Native, Native Hawaii, Multiple-Non-Hispanic)	325	6.8% (5.1–9.1)	172	6.0% (4.3–8.3)	153	7.9% (5.9–10.5)

**Table 2:** Adjusted odds ratios for lifestyle behaviors associated with weight loss intent among adolescent girls (2021 YRBS).

	Weighted *N*	Weighted *%*	Adjusted Odds Ratio (OR) ^1^	95% Confidence Interval (CI)
Dietary intake				
No daily breakfast intake	1951	44.9%	1.00	Reference
Daily breakfast intake	2398	55.1%	0.52	0.40–0.69
No daily vegetable intake (green salad, carrots, or other vegetables excluding potatoes)	3295	76.0%	1.00	Reference
Daily vegetable intake (green salad, carrots, or other vegetables excluding potatoes)	1039	24.0%	0.91	0.77–1.08
No daily green salad intake	4036	92.9%	1.00	Reference
Daily green salad intake	309	7.1%	1.31	0.73–2.35
No daily carrot intake	4158	96.2%	1.00	Reference
Daily carrot intake	166	3.8%	1.01	0.61–1.66
No daily intake of other vegetables (not including salad, carrots, or potatoes)	3500	80.4%	1.00	Reference
Daily other vegetable intake (not including salad, carrots, or potatoes)	854	19.6%	0.78	0.67–0.90
No daily fruit intake	3104	71.3%	1.00	Reference
Daily fruit intake	1247	28.7%	0.91	0.74–1.12
No daily milk intake	722	16.6%	1.00	Reference
Daily milk intake	3626	83.4%	0.90	0.61–1.32
No daily soda intake (not including diet)	3835	88.1%	1.00	Reference
Daily soda intake (not including diet)	517	11.9%	0.99	0.79–1.23
Physical activity				
Less than 3 days per week of muscle-strengthening activities	2939	67.6%	1.00	Reference
Engage in muscle-strengthening activities on 3 days or more per week	1411	32.4%	1.30	1.07–1.57
Less than 5 days per week of 60-min physical activity	2783	62.8%	1.00	Reference
Physically active for 60 min or more on 5 or more days per week	1652	37.2%	1.09	0.82–1.45
Did not play on a sports team	2257	51%	1.00	Reference
Played on 1 or more sports team during the last 12 months	2169	49%	1.11	0.97–1.26
Screen time				
Did not spend 3 or more hours per day using screens	893	20.5%	1.00	Reference
Spent 3 h or more using screens per school day (not including schoolwork) (yes vs. no)	3457	79.5%	1.27	1.02–1.58
Sleep				
Slept less than 8 h	3449	79.3%	1.00	Reference
Slept 8 h or more on an average school night (yes vs. no)	901	20.7%	0.72	0.59–0.89
Substance use				
Did not drink alcohol during the last 30 days	3197	73.5%	1.00	Reference
1 or more drinks of alcohol on one or more days during the last 30 days (yes vs. no)	1153	26.5%	1.61	1.32–1.98
No binge drinking	3872	87.8%	1.00	Reference
Binge drinking (4 or more drinks in a row) on one or more days during the last 30 days (yes vs. no)	539	12.2%	1.29	0.97–1.70
Did not use vapor products during the last 30 days	3414	78.5%	1.00	Reference
Use of electronic vapor product on 1 or more days during the last 30 days (yes vs. no)	936	21.5%	1.22	1.01–1.47
Did not smoke in the last 30 days	4524	96.4%	1.00	Reference
Smoked cigarettes on at least 1 day during the last 30 days	167	3.6%	1.89	0.96–3.72

^1^ Weighted multiple logistic regression models, adjusted for grade level, race/ethnicity, BMI percentile, breakfast intake, sleep, screen time, muscle strengthening activities, alcohol intake, and vaping.

## Data Availability

The data can be downloaded from https://www.cdc.gov/healthyyouth/data/yrbs/data.htm/, accessed on 11 May 2025.

## Abbreviations

The following abbreviations are used in this manuscript:

BMI	Body Mass Index
YRBS	Youth Risk Behavior Surveillance System
CDC	Centers for Disease Control and Prevention
FDA	U.S. Food and Drug Administration
OR	Odds Ratio
CI	Confidence Interval
